# Supplementary material for: Subversion of GBP-mediated host defense by E3 ligases acquired during Yersinia pestis evolution
Source: Nat Commun. 2022 Aug 4;13:4526. doi: 10.1038/s41467-022-32218-y (PMC9352726; doi:10.1038/s41467-022-32218-y)
Supplement: Supplementary file 1 — Supplementary Information [file 41467_2022_32218_MOESM1_ESM.pdf]

Supplementary Information

Yersinia pestis E3 ligases ubiquitinate and degrade GBPs to antagonize the cell-autonomous defense, an arsenal acquired during evolution

Supplementary Figures

Percent Identity

Divergence	1	2	3	4	5	6	7	8	9		
1		38.1	35.4	34.5	34.5	32.0	35.3	30.9	33.9	1	SlrP.pro
2	96.5		68.3	36.8	36.7	36.1	33.5	30.9	34.4	2	SspH1.pro
3	99.3	37.8		40.2	37.1	37.9	34.8	32.3	34.9	3	SspH2.pro
4	110.2	105.3	100.1		69.7	70.6	36.4	30.6	35.7	4	IpH4.5.pro
5	110.1	115.9	113.4	33.4		74.9	36.9	27.8	36.7	5	IpaH9.8.pro
6	112.8	108.3	112.7	33.5	29.4		35.4	26.5	35.0	6	IpaH7.8.pro
7	112.3	103.5	100.2	106.0	101.2	107.3		70.8	83.8	7	YP_3416.pro
8	121.8	100.0	102.8	103.2	100.3	110.1	35.3		76.6	8	YP_3417.pro
9	116.1	104.3	103.4	109.3	102.2	107.1	17.7	25.6		9	YP_3418.pro
	1	2	3	4	5	6	7	8	9		

Supplementary Figure 1. Sequence distances between YspE1/ YspE2 homologs in *Y. pseudotuberculous*, *Shigella* and *Salmonella* bacteria.

Protein sequences shown in Fig. 2b were subjected to multiple sequence alignment by ClustalW using Lasergene software MegAlign and each pairwise distances between all the proteins were calculated.

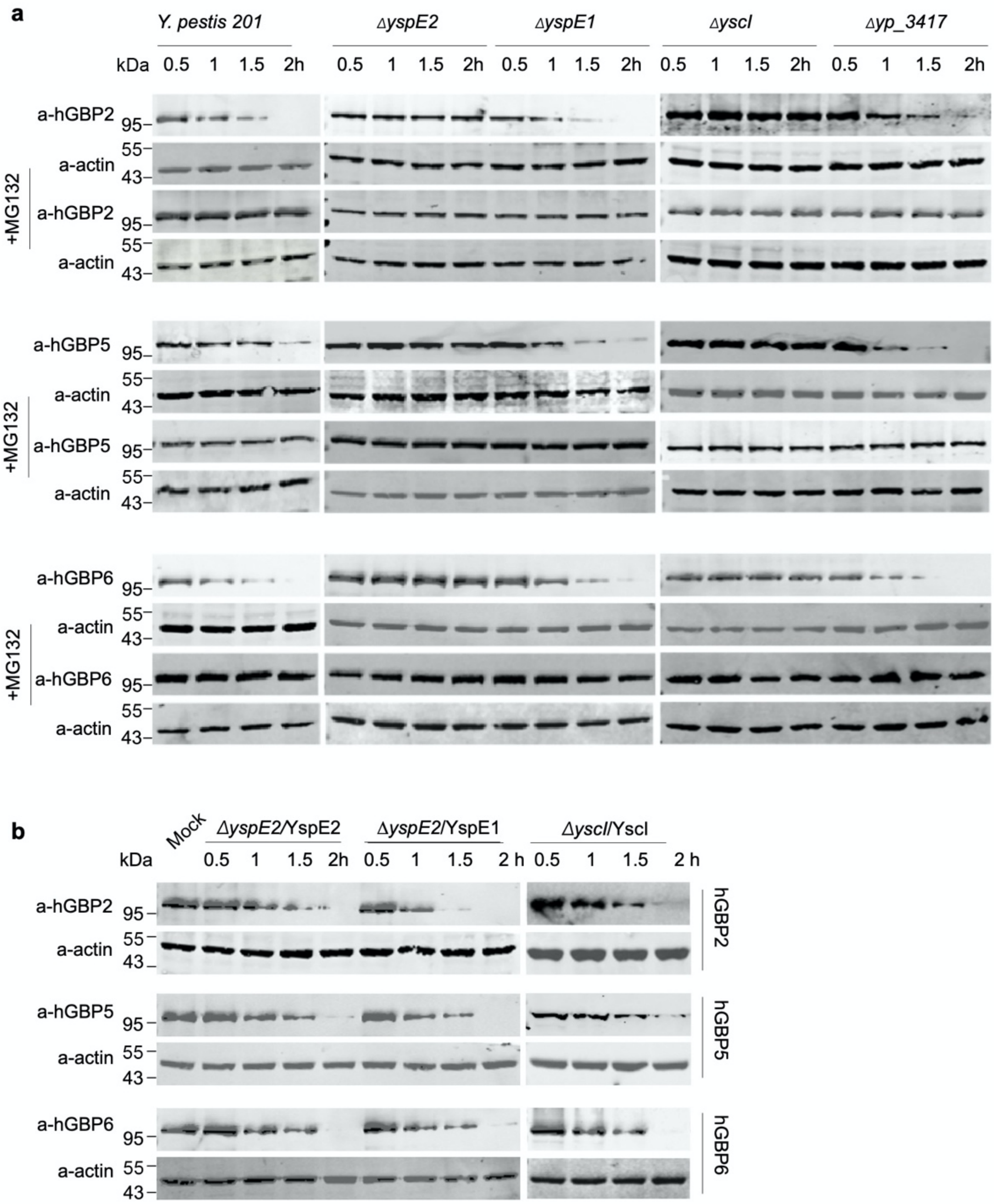

Supplementary Figure 2. *Y. pestis* infections lead to the proteasome degradation of hGBP 2，5 and 6.

(a) HeLa cells stably expressing hGBP 2, 5 and 6 were infected with the wild type *Y. pestis* 201 and various mutants in the presence or absence of MG132 as indicated, and the infected cells were collected at different times and subjected to SDS-PAGE separation and subsequent immunoblotting using specific GBP antibodies. (b) HeLa cells stably expressing hGBP 2, 5 and 6 were infected with *ΔyspE2* complemented with YspE1 or YspE2 expressing plasmid, *ΔyscI* complemented with YscI expressing

plasmid, and the infected cells were analyzed as described in (a). All the experiments have been repeated for three independent times with similar results and only the representative results were shown.

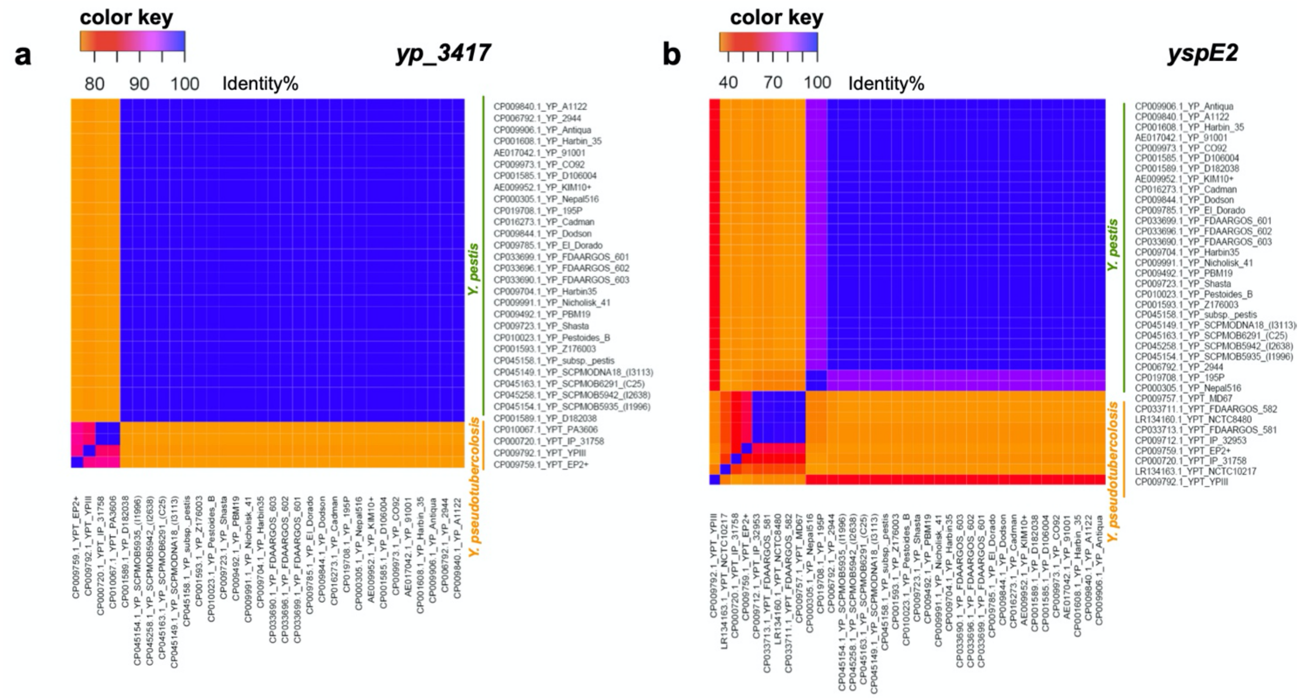

Supplementary Figure 3. Heatmap of intra- and inter species sequence identity for *yp_3417* and *yspE2* among *Y. pseudotuberculosis* and *Y. pestis*.

Heatmap of sequence identity for *yp_3417* (a) and *yspE2* b). The identity values are shown in different colors, with a color scale ranging from orange to blue, with purple and red in between.

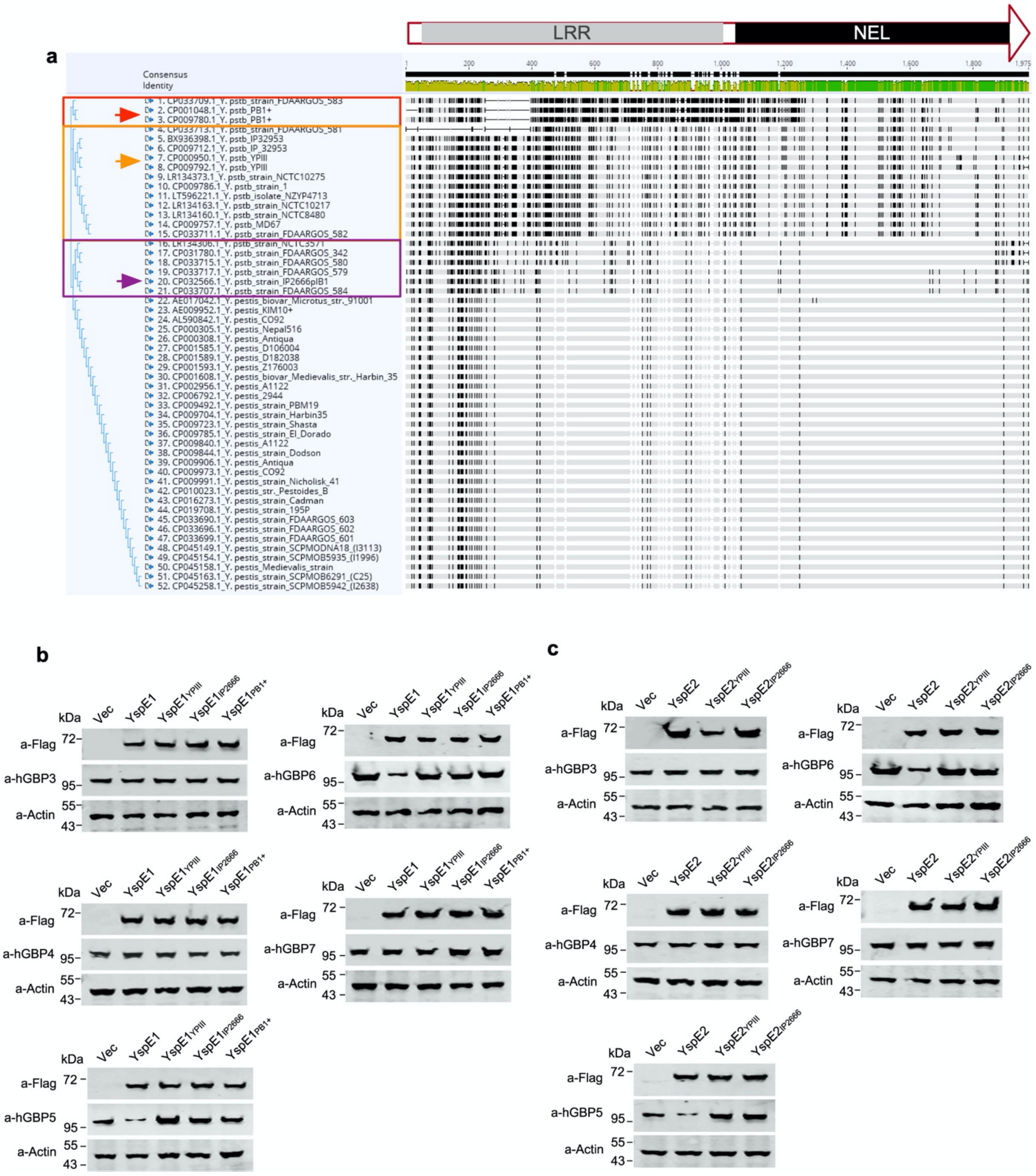

Supplementary Figure 4. YspE1/YspE2 Homologs in *Y. pseudotuberculosis* Diversify Extensively in Sequence and Cannot Degrade hGBPs.

(a) Sequence alignment analysis of YspE1 homologs in *Y. pseudotuberculosis* and *Y. pestis* strains with complete genomes available in the NCBI database. Arrows indicate IP2666pIB1 (purple), YpIII

(orange) and PB1+ (red) strains that represent the three major clusters of YspE1/YspE2 homologs in *Y. pseudotuberculosis*. GBPs degradation activity of YspE1 (b) and YspE2 (c) homologs in *Y. pseudotuberculosis* IP2666pIB1, YPIII and PB1+. Plasmids expressing FLAG-tagged YspE1/YspE2 homologs in three representative *Y. pseudotuberculosis* were individually transfected into IFN-γ treated HeLa cells stably expressing hGBP3-7 as indicated and the transfected cells were lysed and immunoblotted with anti-hGBP antibodies. All the experiments have been repeated for three independent times with similar results and only the representative results were shown.

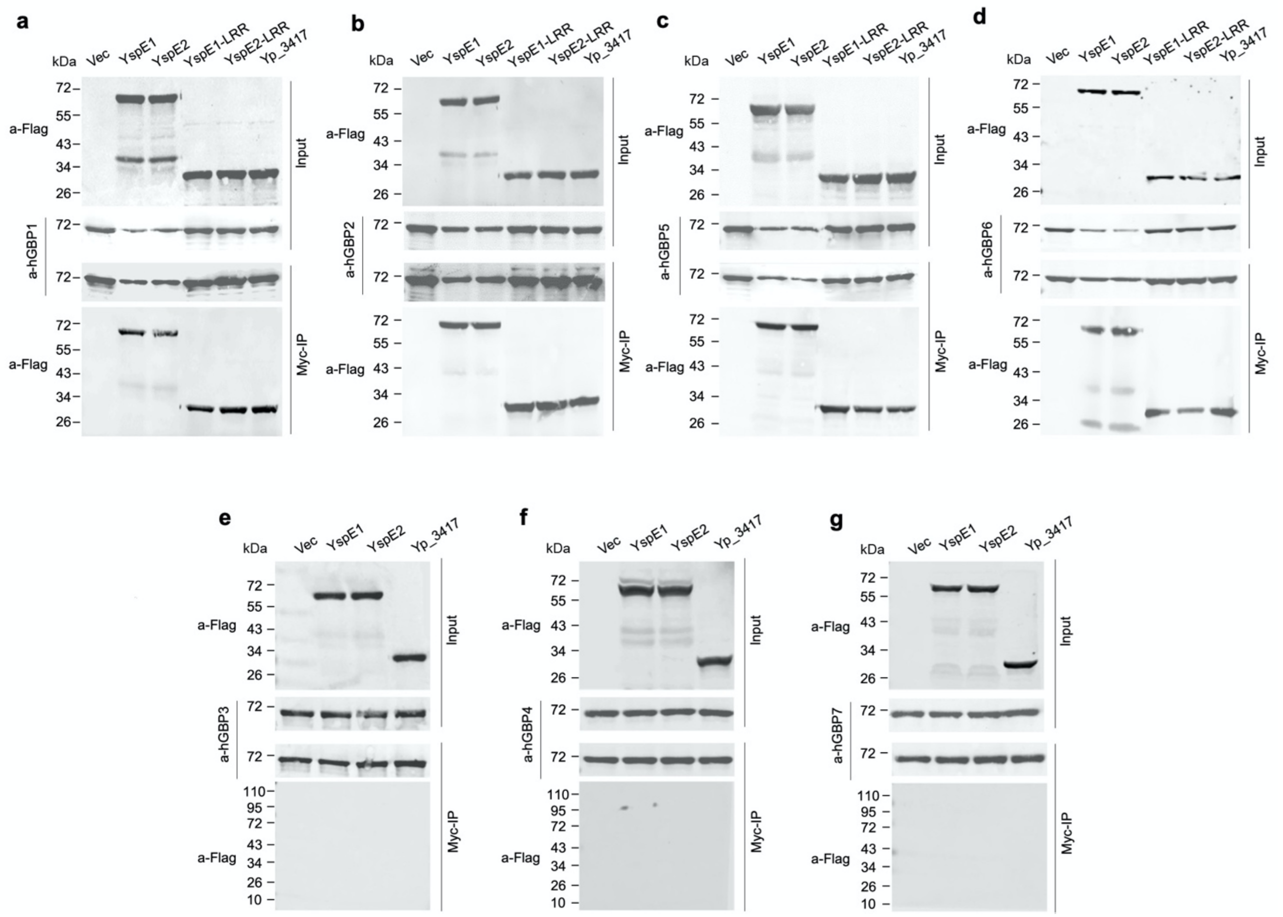

Supplementary Figure 5. Analysis of interactions between YspE1/YspE2 and the different hGBP by coimmunoprecipitation.

Plasmids expressing FLAG-tagged YspE1, YspE2 and their LRR domains, as well as YP_3417, were transiently co-transfected into 293T individually with pCMV-Myc-hGBP1, 2, 5 and 6. Myc-tagged hGBPs were immunoprecipitated with anti-c-Myc beads and the coimmunoprecipitated proteins were measured by immunoblotting analysis using anti-FLAG antibody (a-d). Plasmids expressing FLAG-tagged YspE1, YspE2 and YP_3417 were transiently co-transfected into 293T individually with pCMV-Myc-hGBP 3,4 and 7 (e-g). Co-immunoprecipitation experiments were performed as described for (a-d). All the experiments have been repeated for three independent times with similar results and only the representative results were shown.

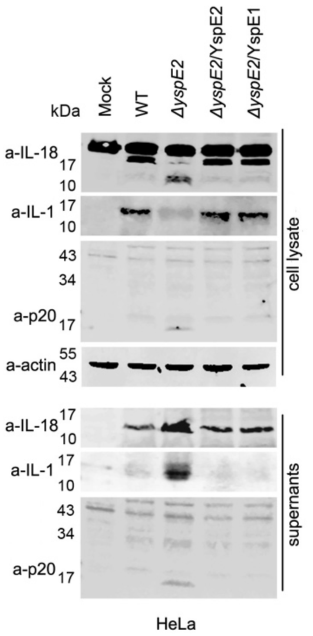

Supplementary Figure 6. Inhibition of the inflammasome activation in HeLa cells by *Y. pestis* requires YspE1/YspE2 E3 ligase activities. HeLa cells were infected with *Y. pestis* 201*, ΔyspE2*, *ΔyspE2*/YspE1, *ΔyspE2*/YspE2 strains as indicated and both the culture medium and cell lysates were analyzed for IL-1β, IL-18 and caspase 1-p20.

Supplementary Tables

Supplementary Table 1. Strains and plasmids used in this study.

Strains or plasmids	Descriptions	Sources
Plasmids		
pDS132	Suicide vector pDS132 used for homologous recombination knockout genes	Laboratory collection
pKD46	Temperature-sensitive plasmid expressing λRed recombinase under the control of arabinose; Apr	Laboratory collection
pUC19-Scarlet	Vector expressing red fluorescent protein	This study
pGEX-4T-2-YspE1	*yp_3416* gene was inserted into pGEX-4T-2; Ap^r	This study
pGEX-4T-2-Yp_3417	*yp_3417* gene was inserted into pGEX-4T-2	This study
pGEX-4T-2-YspE2	*yp_3418* gene was inserted into pGEX-4T-2	This study
pGEX-4T-2-YspE2$_{C386A}$	*yp_3418* gene with Cys-386 to Ala mutation was inserted into pGEX-4T-2	This study
pGEX-4T-2-YspE1$_{C407A}$	*yp_3416* gene with Cys-407 to Ala mutation was inserted into pGEX-4T-2	This study
pGEX-4T-2-YspE1$_{LRR}$	*yp_3416* gene with LRR domain was inserted into pGEX-4T-2	This study
pGEX-4T-2-YspE2$_{LRR}$	*yp_3418* gene with LRR domain was inserted into pGEX-4T-2	This study
pCMV-Myc	Mammalian expression vector expresses proteins containing the N-terminal c-Myc epitope tag.	Addgene
pCMV-Myc-hGBP1	*hGBP1* gene was inserted into pCMV-Myc	This study
pCMV-Myc-hGBP2	*hGBP2* gene was inserted into pCMV-Myc	This study
pCMV-Myc-hGBP3	*hGBP3* gene was inserted into pCMV-Myc	This study
pCMV-Myc-hGBP4	*hGBP4* gene was inserted into pCMV-Myc	This study
pCMV-Myc-hGBP5	*hGBP5* gene was inserted into pCMV-Myc	This study
pCMV-Myc-hGBP6	*hGBP6* gene was inserted into pCMV-Myc	This study
pCMV-Myc-hGBP7	*hGBP7* gene was inserted into pCMV-Myc	This study
Psumo	Expression vectors 6xHis fusion protein; Kana	
pSUMO-hGBP1	*hGBP1* gene was inserted into pSUMO	This study
pSUMO-hGBP4	*hGBP4* gene was inserted into pSUMO	
pACYC184-YspE1	*yp_3416* gene was inserted into pACYC184	This study
pACYC184-YspE2	*yp_3418* gene was inserted into pACYC184	This study
pACYC184-YspE2$_{C386A}$	*yp_3418* gene with Cys-386 to Ala mutation was inserted into pACYC184	This study
pBAD24	Plasmids for expression of the cloned gene under the control of arabinose; Apr	
pBAD24-YscI	*yscI* gene was inserted into pBAD24	
pBAD24-YspE1	*yp_3416* gene was inserted into pBAD24; Ap^r	This study
pBAD24-Yp_3417	*yp_3417* gene was inserted into pBAD24	This study
pBAD24-YspE2	*yp_3418* gene was inserted into pBAD24	This study
pBAD24-YspE1$_{100}$-CyaA	Amino acid 1-100 coding sequences of *yp_3416* gene fusion 1-402 base of *cyaA* gene was inserted into pBAD24	This study
pBAD24-Yp_3417$_{100}$-CyaA	Amino acid 1-100 coding sequences of *yp_3417* gene fusion 1-402 base of *cyaA* gene was inserted into pBAD24	This study
pBAD24-YspE2$_{100}$-CyaA	Amino acid 1-100 coding sequences of *yp_3418* gene fusion 1-402 base of *cyaA* gene was inserted into pBAD24	This study
pBAD24-YopM$_{100}$-CyaA	Amino acid 1-100 coding sequences of *yopM* gene fusion 1-402 base of *cyaA* gene was inserted into pBAD24	This study
pBAD24-CyaA	1-402 base of *cyaA* gene was inserted into pBAD24	This study
p3×Flag-CMV	Mammalian expression vector expresses proteins containing the N-terminal 3×Flag epitope tag.	
pBAD24- -E3YPT$_{IP2666pIB1}$	The coding sequences of xx to xx of IP2666pIB1 inserted into pBAD24	This study
p3×Flag-CMV-YspE1	*yp_3416* gene was inserted into p3×Flag-CMV	This study
p3×Flag-CMV-Yp_3417	*yp_3417* gene was inserted into p3×Flag-CMV	This study
p3×Flag-CMV-YspE2	*yp_3418* gene was inserted into p3×Flag-CMV	This study
p3×Flag-CMV-YspE1$_{LRR}$	*yp_3416* gene with LRR domain was inserted into p3×Flag-CMV	This study

p3×Flag-CMV- YspE2$_{LRR}$	*yp_3418* gene with LRR domain was inserted into p3×Flag-CMV	This study
p3×Flag-CMV-YspE1$_{C407A}$	*yp_3416* gene with Cys-407 to Ala mutation was inserted into p3×Flag-CMV	This study
p3×Flag-CMV-YspE2$_{C386A}$	*yp_3418* gene with Cys-386 to Ala mutation was inserted into p3×Flag-CMV	This study
p3×Flag-CMV-Ypk0744	*Ypk0744* gene was inserted into p3×Flag-CMV	This study
p3×Flag-CMV-Ypk0745	*Ypk0745* gene was inserted into p3×Flag-CMV	This study
p3×Flag-CMV-Ypk0746	*Ypk0746* gene was inserted into p3×Flag-CMV	This study
Strains		
Y. pestis		
201 strain	Wild-type *Y. pestis* strain	
201 strain-Scarlet	201 strain containing plasmid pUC19-Scarlet	This study
201-pBAD24-YspE1$_{100}$-CyaA	201 strain containing plasmid pBAD24-Yp_3416$_{100}$-CyaA	This study
201-pBAD24-YspE2$_{100}$-CyaA	201 strain containing plasmid pBAD24-Yp_3418$_{100}$-CyaA	This study
201-pBAD24-YopM$_{100}$-CyaA	201 strain containing plasmid pBAD24-YopM$_{100}$-CyaA	This study
201-pBAD24-CyaA	201 strain containing plasmid pBAD24-CyaA	This study
201-E3YPT$_{IP2666pIB1}$	*Δyp_3416-18* strain [32] containing plasmid pBAD24- -E3YPT$_{IP2666pIB1}$	This study
ΔyscI	*yscI* gene was replaced by Kanamycin cassette	[35]
ΔyspE1	*yp_3416* gene was eliminated	This study
Δyp_3417	*yp_3417* gene was replaced by Kanamycin cassette	This study
ΔyspE2	*yp_3418* gene was eliminated	This study
ΔyspE1/YspE1	*Δyp_3416* containing plasmid pACYC184-Yp_3416	This study
ΔyspE2/YspE1	*Δyp_3418* containing plasmid pACYC184-Yp_3416	This study
ΔyspE2/YspE2	*Δyp_3418* containing plasmid pACYC184-Yp_3418	This study
ΔyspE2/YspE1$_{C386A}$	*Δyp_3418* containing plasmid pACYC184-Yp_3418$_{C386A}$	This study
ΔyscI-pBADYscI	*ΔyscI* containing plasmid pBAD24-YscI	[35]
ΔyspE1-pBAD3416	*Δyp_3416* containing plasmid pBAD24-Yp_3416	This study
Δyp_3417-pBAD3417	*Δyp_3417* containing plasmid pBAD24-Yp_3417	This study
ΔyspE2-pBAD3418	*Δyp_3418* containing plasmid pBAD24-Yp_3418	This study
ΔyspE2-Scarlet	*Δyp_3418* containing plasmid pUC19-Scarlet	This study
ΔyspE2-Yp_3418-Scarlet	*Δyp_3418-pACYC184-Yp_3418* containing plasmid pUC19-Scarlet	This study
Δyp_3418-Yp_3418$_{C386A}$-Scarlet	*Δyp_3418-pACYC184-Yp_3418C$_{386A}$* containing plasmid pUC19-Scarlet	This study
Y. pseudotuberculosis		
PA3606	Wild-type *Y. pseudotuberculosis* strain	Laboratory collection
E. coli S17-1λpir	Used to construct a seamless knockout strain of *Y. pestis*	

Supplementary Table 2. 38 *Y. pestis* strains with complete genomes available in the NCBI database (sequences were download on Oct 10th, 2020) were subjected to multiple sequences alignment analysis in this study.

Y. pestis Strains	Accessions	Branch	presence of *yp_3416~3418* loci
1412	CP006783.1	0.PE2	No
1413	CP006762.1	0.PE2	No
1522	CP006758.1	0.PE2	No
3067	CP006754.1	0.PE2	No
3770	CP006751.1	0.PE2	No
8787	CP006748.1	0.PE2	No
Angola	CP009935.1	0.PE3a	No
Pestoides F	CP009715.1	0.PE2	No
SCPM-O-B-6899 (231)	CP045145.1	0.ANT3	No
Pestoides G	CP010247.1	0.PE2	No
FDAARGOS_602	CP033696.1	0.PE4B	Yes
195/P	CP019708.1	2.ANT1	Yes
2944	CP006792.1	2.MED1	Yes
91001	AE017042.1	0.PE4	Yes
A1122	CP009840.1	1.ORI1	Yes
Antiqua	CP009906.1	1.ANT	Yes
Cadman	CP016273.1	1.ORI1	Yes
CO92	CP009973.1	1.ORI1	Yes
D106004	CP001585.1	1.IN4	Yes
D182038	CP001589.1	1.IN3	Yes
Dodson	CP009844.1	1.ORI1	Yes
El Dorado	CP009785.1	1.ORI1	Yes
FDAARGOS_601	CP033699.1	1.ANT	Yes
FDAARGOS_603	CP033690.1	2.ANT3	Yes
Harbin 35	CP001608.1	2.ANT3	Yes
Harbin35	CP009704.1	2.ANT3	Yes
KIM10+	AE009952.1	2.MED1	Yes
Nepal516	CP000305.1	2.ANT1	Yes
Nicholisk 41	CP009991.1	2.ANT3	Yes
PBM19	CP009492.1	1.ORI2	Yes
Pestoides B	CP010023.1	0.PE4	Yes
SCPM-O-B-5935 (I-1996)	CP045154.1	2.ANT3	Yes
SCPM-O-B-5942 (I-2638)	CP045258.1	4.ANT1	Yes
SCPM-O-B-6291 (C-25)	CP045163.1	2.MED1	Yes
SCPM-O-B-6530	CP045158.1	2.MED0	Yes
SCPM-O-DNA-18 (I-3113)	CP045149.1	4.ANT1	Yes
Shasta	CP009723.1	1.ORI1	Yes
Z176003	CP001593.1	1.IN2	Yes

Supplementary Table 3. 23 *Y. pseudotuberculosis* strains with complete genomes available in the NCBI database (sequences were download on Oct 10th, 2020) were subjected to multiple sequences alignment analysis in this study.

***Y. pseudotuberculosis* Strains**	**Accessions**
PA3606	CP010067.1
FDAARGOS_665	CP044064.1
ATCC 6904	CP008943.1
IP 31758	CP000720.1
EP2/+	CP009759.1
PB1/+	CP009780.1
IP 32953	CP009712.1
Y. pseudotuberculosis strain 1	CP009786.1
NZYP4713	LT596221.1
FDAARGOS_580	CP033715.1
FDAARGOS_579	CP033717.1
FDAARGOS_342	CP031780.1
MD67	CP009757.1
IP2666pIB1	CP032566.1
FDAARGOS_584	CP033707.1
FDAARGOS_583	CP033709.1
FDAARGOS_582	CP033711.1
FDAARGOS_581	CP033713.1
NCTC10217	LR134163.1
NCTC8480	LR134160.1
NCTC3571	LR134306.1
YPIII	CP000950.1
NCTC10275	LR134373.1
