# Supplementary material for: Subversion of GBP-mediated host defense by E3 ligases acquired during Yersinia pestis evolution
Source: Nat Commun. 2022 Aug 4;13:4526. doi: 10.1038/s41467-022-32218-y (PMC9352726; doi:10.1038/s41467-022-32218-y)
Supplement: Supplementary file 3 — Description of Additional Supplementary Information [file 41467_2022_32218_MOESM3_ESM.pdf]

Description of Additional Supplementary Information

Supplementary Movies 1-4.

Live microscopy analysis of GFP-hGBP1 signals in HeLa cells infected with the various Y. pestis strains. During infection, images were taken every 5 mins until 8 hours of infection using spinning disk system with Nikon ECLIPSE Ti-E microscopy.

Title: Supplementary Movie 1

Description: HeLa cells stably expressing hGBP1 were seeded in Lab-Tek chambered cover-glass and infected with the wild type Y. pestis

Title: Supplementary Movie 2

Description: HeLa cells stably expressing hGBP1 were seeded in Lab-Tek chambered cover-glass and infected with the wild type ΔyspE2

Title: Supplementary Movie 3

Description: HeLa cells stably expressing hGBP1 were seeded in Lab-Tek chambered cover-glass and infected with the wild type ΔyspE2/YspE2

Title: Supplementary Movie 4

Description: HeLa cells stably expressing hGBP1 were seeded in Lab-Tek chambered cover-glass and infected with the wild type ΔyspE2/YspE2C386A
