# Supplementary material for: Subversion of GBP-mediated host defense by E3 ligases acquired during Yersinia pestis evolution
Source: Nat Commun. 2022 Aug 4;13:4526. doi: 10.1038/s41467-022-32218-y (PMC9352726; doi:10.1038/s41467-022-32218-y)
Supplement: Supplementary file 8 — Reporting Summary [file 41467_2022_32218_MOESM8_ESM.pdf]

nature research

Corresponding author(s): Zongmin Du, Ruifu Yang

Last updated by author(s): Jun 30, 2022

Reporting Summary

Nature Research wishes to improve the reproducibility of the work that we publish. This form provides structure for consistency and transparency in reporting. For further information on Nature Research policies, see our Editorial Policies and the Editorial Policy Checklist.

Statistics

For all statistical analyses, confirm that the following items are present in the figure legend, table legend, main text, or Methods section.

n/a	Confirmed	
☐	☒	The exact sample size (*n*) for each experimental group/condition, given as a discrete number and unit of measurement
☒	☐	A statement on whether measurements were taken from distinct samples or whether the same sample was measured repeatedly
☐	☒	The statistical test(s) used AND whether they are one- or two-sided<br>*Only common tests should be described solely by name; describe more complex techniques in the Methods section.*
☒	☐	A description of all covariates tested
☐	☒	A description of any assumptions or corrections, such as tests of normality and adjustment for multiple comparisons
☐	☒	A full description of the statistical parameters including central tendency (e.g. means) or other basic estimates (e.g. regression coefficient) AND variation (e.g. standard deviation) or associated estimates of uncertainty (e.g. confidence intervals)
☐	☒	For null hypothesis testing, the test statistic (e.g. *F*, *t*, *r*) with confidence intervals, effect sizes, degrees of freedom and *P* value noted<br>*Give P values as exact values whenever suitable.*
☒	☐	For Bayesian analysis, information on the choice of priors and Markov chain Monte Carlo settings
☒	☐	For hierarchical and complex designs, identification of the appropriate level for tests and full reporting of outcomes
☐	☒	Estimates of effect sizes (e.g. Cohen's *d*, Pearson's *r*), indicating how they were calculated

Our web collection on statistics for biologists contains articles on many of the points above.

Software and code

Policy information about availability of computer code

Data collection	Confocal images were acquired using an Andor Dragonfly spinning disk confocal microscope.<br>Living images were taken every using spinning disk system with Nikon ECLIPSE Ti-E microscopy.<br>SPR measurements were performed on a Biacore T200 instrument (GE Healthcare).
Data analysis	The software MEGA (v7.0.26) was used to do multiple sequences alignment and compute pairwise distances.<br>Data of real-time monitored SPR assays was analyzed using the Biacore T200 Evaluation Software 2.0.<br>Statistical analysis and survival curves was performed using GraphPad Prism 8.0.<br>Protein sequences shown in Fig. 2B were subjected to multiple sequence alignment using DANSTAR Lasergene V7.1.0.<br>Gene homologs identity between two sequences were displayed using the package of heatmap in R (v3.6.1)

For manuscripts utilizing custom algorithms or software that are central to the research but not yet described in published literature, software must be made available to editors and reviewers. We strongly encourage code deposition in a community repository (e.g. GitHub). See the Nature Research guidelines for submitting code & software for further information.

Data

Policy information about availability of data

All manuscripts must include a data availability statement. This statement should provide the following information, where applicable:

- Accession codes, unique identifiers, or web links for publicly available datasets
- A list of figures that have associated raw data
- A description of any restrictions on data availability

All data generated or analyzed during this study are included in this published article and supplementary information files. Source data are provided with this paper. Sequences of Y. pestis and Y. pseudotubercolusis strains used in this study were download from NCBI database (https://www.nlm.nih.gov/assembly).

Field-specific reporting

Please select the one below that is the best fit for your research. If you are not sure, read the appropriate sections before making your selection.

☒ Life sciences ☐ Behavioural & social sciences ☐ Ecological, evolutionary & environmental sciences

For a reference copy of the document with all sections, see nature.com/documents/nr-reporting-summary-flat.pdf

Life sciences study design

All studies must disclose on these points even when the disclosure is negative.

Sample size	No statistical methods were used to predetermine sample size. For animal experiments, groups (n=10) of female mice were challenged with bacteria suspensions for both the survive curve experiments and the bacterial burden analysis. Both previously published works and our studies showed that 10 mice per group were sufficient to overcome the effects of individual variations and get reproducible results. For other experiments that involves cell lines or BMDMs, a minimum of three technical replicates were used per sample, and experiments were repeated multiple times independently.
Data exclusions	No technical replicates were excluded from the analysis.
Replication	All the experiments have been repeated at least three times, except that the bacterial loads in mice were repeated for two independent times and a representative result was presented in this study.
Randomization	All the experiments used the common cells for different groups. For animal experiments, all the female mice at 6-8 weeks were randomly grouped.
Blinding	All the data were collected by equipments automatically and analyzed quantitatively according to a common criteria across all samples..

Reporting for specific materials, systems and methods

We require information from authors about some types of materials, experimental systems and methods used in many studies. Here, indicate whether each material, system or method listed is relevant to your study. If you are not sure if a list item applies to your research, read the appropriate section before selecting a response.

Materials & experimental systems

n/a	Involved in the study	
☐	☒	Antibodies
☐	☒	Eukaryotic cell lines
☒	☐	Palaeontology and archaeology
☐	☒	Animals and other organisms
☒	☐	Human research participants
☒	☐	Clinical data
☒	☐	Dual use research of concern

Methods

n/a	Involved in the study	
☒	☐	ChIP-seq
☒	☐	Flow cytometry
☒	☐	MRI-based neuroimaging

Antibodies

Antibodies used	Antibody (catalog number): manufacturer, dilution(s)<br>-Caspase-1 p20 (#PA5-94989): Thermo Fisher Scientific (Waltham, MA, USA), 1:1000 WB<br>-IL-18 (#PA5-79479) and IL-1β (#701304) antibodies: Thermo Fisher Scientific (Waltham, MA, USA), 1:1000 WB<br>-Ubiquitin linkage-specific K48 (ab140601) and Ubiquitin linkage-specific K63 (ab179434) antibodies: Abcam (Cambridge, UK), 1:2000 WB<br>-hGBP1 (ab121039), hGBP2 (ab203238), hGBP3 (ab74061), hGBP4 (ab173697), hGBP5 (ab130569), hGBP6 (ab125704), hGBP7 (ab104293) antibodies: Abcam (Cambridge, UK), 1:2000 WB

	-β-actin (RM2001), c-Myc-Tag (KM8003), Flag-Tag (KM8002) antibodies: Sungene Biotech (Tianjin, China), 1:5000 WB<br>-Ubiquitin (A-100): Boston Biochem (Cambridge, MA, USA), 1:2000 WB<br>-GFP-Tag (314487): Abmart (Shanghai, China), 1:5000 WB<br>-IRDye 800CW-conjugated goat anti-rabbit antibody (C90529-19) and IRDye 800CW-conjugated goat anti-mouse antibody (C81106-03): LI-COR Biosciences (Lincoln, NE, USA), 1:10000 WB<br>-YP_3416, YP_3418 and YopM antibodies were rabbit polyclonal antibodies prepared by our laboratory, 1:200 WB<br>WB - western blot
Validation	All the antibodies used in this study have been validated by showing acceptable signals in immunoblotting analysis in the corresponding positive samples and no obvious unspecific bands interfering the results.<br>Antibody (catalog number): validation<br>-Caspase-1 p20 (#PA5-94989): WB & IF<br>-IL-18 (#PA5-79479) and IL-1β (#701304): WB & IF<br>-Ubiquitin linkage-specific K48 (ab140601) and Ubiquitin linkage-specific K63 (ab179434): WB & IF<br>-hGBP1 (ab121039), hGBP2 (ab203238), hGBP3 (ab74061), hGBP4 (ab173697), hGBP5 (ab130569), hGBP6 (ab125704), hGBP7 (ab104293): WB<br>-β-actin (RM2001), c-Myc-Tag (KM8003) and Flag-Tag (KM8002): WB & IF<br>-Ubiquitin (A-100): WB<br>-GFP-Tag (314487): WB<br>-IRDye 800CW-conjugated goat anti-rabbit antibody (C90529-19) and IRDye 800CW-conjugated goat anti-mouse antibody (C81106-03): WB<br>-YP_3416, YP_3418 and YopM: WB<br>WB - western blot, IF - immunofluorescence

Eukaryotic cell lines

Policy information about cell lines

Cell line source(s)	HeLa cells stably expressing hGBP1~7 were obtained from Dr Feng Shao's lab (National Institute of Biological Sciences, Beijing, China)<br>HeLa, RAW264.7, 293T and U937 cells used in this study were obtained from the American Type Culture Collection (ATCC).
Authentication	HeLa cell lines stably expressing hGBP1-7 have been described in a previously published paper (Li, P., et al. Ubiquitination and degradation of GBPs by a Shigella effector to suppress host defence. Nature 551, 378-383).<br>All the cells used were checked by their morphological features but have not authenticated.
Mycoplasma contamination	Cell lines were tested negative for mycoplasma.
Commonly misidentified lines<br>(See ICLAC register)	No commonly misidentified cell line was used.

Animals and other organisms

Policy information about studies involving animals; ARRIVE guidelines recommended for reporting animal research

Laboratory animals	Female C57 BL/6J mice at 6-8 weeks (wild-type or Gbpchr3-/,-chr5-/--) were used in this study. Wild-type mice were obtained from Vitalriver Company (Beijing, China) and Gbpchr3-/,-chr5-/-- mice were generated in-house as reported in the Methods.
Wild animals	The study did not involve wild animals.
Field-collected samples	The study did not involve samples collected from the field.
Ethics oversight	Animals were handled in strict accordance to the Guidelines for the Welfare and Ethics of Laboratory Animals of China and all the animal experiments were approved by the Institutional Animal Care Committee of Beijing Institute of Epidemiology and Microbiology (protocol IACUC-DWZX-2020-062)

Note that full information on the approval of the study protocol must also be provided in the manuscript.
